# Reproductive Technologies and Genomic Selection in Cattle

**DOI:** 10.4061/2010/192787

**Published:** 2010-10-24

**Authors:** Patrice Humblot, Daniel Le Bourhis, Sebastien Fritz, Jean Jacques Colleau, Cyril Gonzalez, Catherine Guyader Joly, Alain Malafosse, Yvan Heyman, Yves Amigues, Michel Tissier, Claire Ponsart

**Affiliations:** ^1^UNCEIA, Department of Research and Development, 13 rue Jouet, 94704 Maisons Alfort, France; ^2^Department of Clinical Studies, SLU, 750-07 Uppsala, Sweden; ^3^UNCEIA Dpt Fédéral, 75595 Paris, France; ^4^INRA UMR1313 GABI, 78352 Jouy en Josas, France; ^5^UNCEIA, Department of Research and Development, 38300 Chateauvillain, France; ^6^INRA BDR, 78350 Jouy en Josas, France; ^7^LABOGENA, 78350 Jouy en Josas, France; ^8^UMOTEST, 01250 Cezeyriat, France

## Abstract

The recent development of genomic selection induces dramatic changes in the way genetic selection schemes are to be conducted. This review describes the new context and corresponding needs for genomic based selection schemes and how reproductive technologies can be used to meet those needs. Information brought by reproductive physiology will provide new markers and new improved phenotypes that will increase the efficiency of selection schemes for reproductive traits. In this context, the value of the reproductive techniques including assisted embryo based reproductive technologies (Multiple Ovaluation Embryo Transfer and Ovum pick up associated to *in vitro* Fertilization) is also revisited. The interest of embryo typing is discussed. The recent results obtained with this emerging technology which are compatible with the use of the last generation of chips for genotype analysis may lead to very promising applications for the breeding industry. The combined use of several embryo based reproductive technologies will probably be more important in the near future to satisfy the needs of genomic selection for increasing the number of candidates and to preserve at the same time genetic variability.

## 1. Introduction

During recent decades, advancement in our knowledge of reproductive physiology and improvements in embryo-based reproductive biotechnologies have facilitated the development of a rather complete “tool box” including reproductive techniques used either for commercial purposes and/or in the frame work of breeding schemes. These techniques currently have varying degrees of efficiency [1] and for most of them continuous improvements may be expected in the future. Used alone or in combination, their development is influenced in many different ways including ethics and general acceptance, consumer demand for specific products, regulatory changes, and also changes related to the evolution of breeding strategies. 

The recent development of genomic selection has led to dramatic changes in the way genetic selection schemes are to be conducted [2, 3]. Due to the present and expected evolution in the organisation of selection strategies and associated requirements, the value of the various reproductive techniques used today for commercial purposes and in genetic schemes should be revisited.

## 2. The New Context and Corresponding Needs for Genomic-Based Selection Schemes

In genetic selection, the expression for a given trait is the phenotype that integrates the effect of genes and the effect of environmental factors. In the past, the effect of the genetic component was evaluated from genealogy and by measuring performances/phenotyping of candidates or of their progeny. Today, in association with information issued from genealogy, genomic selection, while linking from previous experience, the presence of genes and/or polymorphism of those genes to performances allows to predict the genetic value of a candidate which is revealed by the presence of pertinent markers indicative of its genotype. 

In France, Marker Assisted Selection (MAS) has been developed since 10 years and was based initially on a limited number of micro satellite analyses for a few Quantitative Trait Loci (QTL). Selection was performed by combining this first generation of genomic information with conventional indexes arising from quantitative genetics. Further developments followed the work of Meuwissen et al. [4] who have shown that it was possible to predict the total genetic value of animals or plants by using genome-wide dense marker maps. The progress of the knowledge of the bovine genome and of DNA analyses has made dense marker maps available in this species and the position of markers in relation to genes of interest has been refined. This allows animal breeding companies to use today sets of thousands of genetic markers to select animals [5–10]. The development of genomic techniques will probably make available the use of the complete genome information for selection purposes in a few years [11]. Different types of chips based on the use of Single Nucleotide Polymorphism (SNP, i.e., a single base difference on the DNA between individuals or groups of individuals) are available and can be used to achieve different objectives. The bovine 50 K SNP chip has become the standard tool for breeding industries in dairy cattle, and a higher density chip 800 K SNP is also available to screen for more genes and for a deeper implementation of genomic selection. A smaller and less expensive 3 K SNP chip is now also available to screen large populations [11]. Today, a lot of progress has been achieved for the Holstein breed and in other major dairy breeds (such as the Montbeliarde and Normande breeds) in which all the characters previously evaluated using classical selection by quantitative genetics can now be evaluated from genomic information [12]. For instance, in France, recent developments allow this evaluation to be made by using several hundreds of markers per character instead of 30 QTL per characters as in the previous MAS evaluation [6]. Due to those technical improvements, application to small breeds can now be expected [5] and efforts should be made to also get appropriate phenotypic information for those breeds which have not been studied as intensively as the Holstein breed and other major dairy breeds.

In parallel with those technological changes, attempts have been made in different countries to reinforce the value of the genomic information by including more and more animals in the evaluation and selection process [5, 7]. Consequently, more reliable estimates can be obtained for the desired traits while genetic variability is better preserved. Candidates will have to be produced from parents of different pedigree's (maximum of families within a breed) and at the same time breeding should be organised in a way to maximize the variability of the next generation. The potential advantages of genomic selection programmes run on these principles have been shown recently by Monte-Carlo simulations on full-size breeding schemes [13]. This work demonstrated that by multiplying the number of candidates by 3 it was possible at the same time to increase genetic progress dramatically (+80% when compared to the classical breeding scheme) while decreasing inbreeding rate (−23%). 

**Figure 1:**
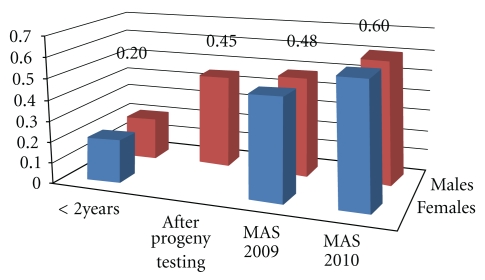
Accuracy of fertility Estimated Breeding Value of young animals (<2 years of age) and after progeny test without molecular information, and of animals with MAS information (MAS 2009, MAS 2010) obtained before 2 years of age [12, 14].

Due to its costs and to the fact that the genetic value of a given future sire is known with enough precision from genomic analyses, the need for progeny testing will be considerably reduced or even removed [3]. For some traits, such as those related to fertility, the precision associated with genomic indexes is or will be much better than with classical selection [12, 14] (Figure 1).

Questions are raised about the need to keep a common reference basis in different populations to optimize the evaluation process and evaluate the changes induced by genomic selection. This is illustrated by the recent agreement in the consortium “Eurogenomics”, a group of breeding companies from France, The Netherlands, Germany, Denmark, Finland, and Sweden to gather and share the genomic information for evaluation of breeding values from a common reference basis including 16 000 Holstein sires [15].

Research is made also on computational methodologies to define the best way to analyse and use the huge quantity of information arising from genomic analyses of a very large number of animals [5].

For all traits of interest, these changes highlight the *importance of the phenotypic information* that must be unified from large number of animals in the reference base and which becomes one of the main bottlenecks in the process.

## 3. How Can Reproductive Physiology and Reproductive Techniques Help to Meet the Needs of Genomic Selection and Commercial Production within this Context?

### 3.1. Reproductive Physiology

In an attempt to improve numerous traits by genomic selection, knowledge of the relationships between genome information and phenotypic criteria is of crucial importance. Following initial studies [16–19], the development of microarrays (dedicated or generic) helped to characterise the relationships between genotype and phenotype. More recently, high throughput technologies for DNA sequencing and RNA analysis have become more and more affordable and are now currently used in research programs aiming to study relationships between genotype and phenotype and gene expression. With such objectives, phenotyping (animal models, precise criteria, and methods) becomes the main bottleneck to achieve this goal. As a consequence, there will be a need for research aiming to phenotype new critical traits and/or to improve the precision of the phenotypes for existing traits. For instance, for reproductive traits, it is possible, by using measurements of progesterone and pregnancy associated proteins, to characterise relatively well pregnancy failure [20]. This has been used in a genomic programme aiming to screen (from a large database) for the existence of candidate mutations that may explain differences in fertility between progeny groups [21]. However, for such an approach, methods allowing one to distinguish fertilization failure from early embryonic mortality are still lacking and such developments would be most valuable to find new markers of fertility. Similarly, a lot of information and new physiological markers for fertility may be derived from investigations made to better characterize reproductive function. For instance, due to the strong relationships existing between oocyte growth and maturation and subsequent embryonic development [22], programs aiming to study links between follicular growth, oocyte quality, and the presence of genomic markers by using proteomics, lipidomics, and metabolomics may be particularly appropriate to find new markers for fertility [23].

### 3.2. Use of Embryo-Based Biotechnologies

One of the most important features of the new selection procedures will be to considerably increase the number of candidates submitted to genomic selection to maximize the chances of getting interesting individuals that will be positively evaluated for a large number of traits. As mentioned before, this will allow an increase in the selection pressure for those traits. Also, it will be possible to use bulls for AI at a younger age, thereby lowering the generation interval. Finally, the use of groups of bulls with a favourable genomic index will improve the precision of indexes when compared to the use of a very limited number of older sires as was the case in the past. This may be also favourable to genetic variability if adequate and wise breeding schemes are implemented; otherwise shortening the generation interval may also lead to an increased inbreeding rate. 

The way to produce these large numbers of animals becomes critical. In this context, AI alone may be inadequate to generate sufficient animals in a given period of time and the efficiency of MOET and OPU-IVP looks more and more critical to produce these large numbers of animals to be genotyped. With these “intensive” embryo-based reproductive techniques, it is relatively easy to increase the number of candidates by increasing the number of flushes in MOET schemes. When compared to MOET, the number of embryos produced in a given period of time can even be multiplied by 2 or 3 [1, 24] by the use of repeated OPU-IVF sessions leading to the production of approximately 70 calves/donor and per year. Females of various origins can be collected to preserve genetic variability and this technique presents additional advantage if different bulls are used for different OPU sessions or even within a session [1, 24].

A lot of research has been done to improve in vitro culture systems to attempt to mimic as much as possible the oviduct fluid environment. Synthetic Oviduct Fluid (SOF) based culture systems are the most commonly used today and overall development rates to the blastocyst stage of 30–40% are achieved by most teams. This is probably as much as can be expected given the heterogeneity in follicles typically used. The effect of a previous superovulation on fertilisation and subsequent embryonic development is still controversial as some authors reported detrimental effects [25, 26] whereas other studies show similar embryonic development rates under those 2 conditions [1]. Irrespective of the type of treatment of the donor female and type of culture system, it has been shown very clearly from most studies that there is a significant decrease in embryo production when oocytes are matured in vitro in standard medium compared to in vivo conditions [25–29]. This emphasizes the roles of the final steps of oocyte growth and maturation in subsequent embryo development which have also been illustrated by epidemiological studies made under in vivo conditions showing relationships between certain factors influencing these steps and embryonic mortality [22]. There is probably a lot of progress that can be achieved in in vivo and in vitro embryo production by optimizing the conditions under which the oocytes are growing within follicles in donor females. Handling at the time of collection and thereafter as well as in vitro maturation are also critical steps to be optimized since dramatic metabolic changes occur very quickly after oocyte recovery [30]. 

Despite the above-mentioned limitations and potential margins for progress, the work that has been done in the past 15 years to improve oocyte collection and in vitro embryo production systems has made those systems viable and practically useful by the most advanced breeding companies to produce more embryos in their genetic schemes [24, 31]. However, (i) to mismanage the use of these techniques may lead to increase inbreeding significantly especially if bull dams are overexploited (Colleau 2010, personal communication) and (ii) due to the new requirements in relation to the implementation of genomic selection (especially those related to the increase in the number of candidates), additional strong limitations exist for giving birth to a very large number of calves that would be genotyped after birth. 

Effectively, one of the main bottlenecks experienced by breeding organisations working in Europe with dairy cattle is the limited availability of female recipients. This is reinforced by the fact that, due to lower pregnancy rates when using cows instead of heifers as recipients, the efficiency of embryo transfer is much lower if the heifers are used mainly as donors and not as recipients [1]. In addition to this, high costs will be induced by the transfer of a very large number of embryos into recipients that must be maintained pregnant until birth of progeny and the economic potential of the nonselected calves will be low. When producing these candidate animals on farm, the amount of field work in relation to embryo transfer and in vitro production will be even greater than today and will generate high logistical costs. Finally, this process may increase the contractual cost with individual farmers especially due to the potential existence of very interesting candidates identified by genomics. 

For these reasons, genotyping the embryos and selecting them before transfer appears to be an attractive scenario to maximize the chances to finding interesting individuals for multiple traits while transferring a “reasonable” number of embryos.

### 3.3. Embryo Typing

The interest of embryo typing for breeding companies was discussed even before the emergence of the new techniques for genomic selection that includes today thousands of markers [24]. As soon as MAS based on a limited number of micro satellites could be used, advantages were found due to its potential value for screening the embryo for several traits. At the same time, embryo sexing could be used at a very low cost during the process of genomic analyses. Doing typing and selection early in life was also expected to be a solution to shorten the generation interval and to limit the costs of producing the high number of calves and associated costs of the existing progeny testing to achieve multicharacter selection. Today the potential advantages of combining intensive embryo production and genotyping are even higher. 

Results reported initially in the literature for ruminants [32, 33] were based on the typing for a limited number of markers. Peippo et al. [32] have shown that it was possible to genotype embryonic biopsies for a limited number of micro satellites and to get subsequent pregnancies after transfer of the corresponding biopsied bovine embryos. Similarly, in the goat, Guignot et al. [33] reported the possible use of embryo genotyping for a very limited set of markers to screen for sensitivity to scrapie combined with sex determination. The most recent published results emerged from the programme “TYPAGENAE” in which the efficiency of embryo typing was tested from a set of 45 micro satellites corresponding to the first generation of MAS [34–36]. 

In a first step, in vitro produced embryos were used to assess the accuracy and repeatability of embryo-based genotyping. Day 6 embryos were biopsied and each blastomere from the biopsy was submitted to embryonic cloning to reconstitute full blastocysts [37]. A mean of 2 full blastocysts were obtained from cloning blastomeres and more than 95% of the embryos survived in culture following biopsy. The results of typing obtained from the reconstituted blastocyst and the donor embryo were subsequently compared from a total of 41 samples. The proportion of successfully typed samples was >90%. The typing of the cloned embryos corresponded all the time to the typing of the original embryos and genotypes were fully compatible with the genotypes of the parents. The error rate, when considering differences between the different types of samples, was 3% and all errors were due to the lack of identification of one of the alleles (drop-out).

**Figure 2:**
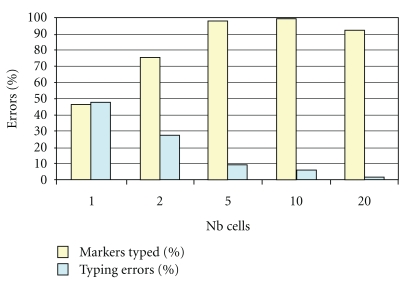
Effect of the number of cells of the biopsy on the percentage of detection of microsatellites (markers typed) and on the percentage of typing errors [2, 36].

From a second series of experiments, the typing results between biopsies of 10–20 cells performed at the blastocyst stage and the rest of the embryo were compared. Whole Genome Amplification (WGA) was applied on cell extracts from the biopsy before typing. From 60 samples, 95% were genotyped and a similar rate of allelic drop out was observed when compared to analyses made from full embryos (2-3%). Another set of 40 samples was used to evaluate the minimum number of cells to be biopsied before pre amplification. WGA was performed on all samples and allowed genotyping in 98% of cases with <10% drop out rates from biopsies of 8–10 cells. This rate was much higher in biopsies containing less than 5 cells [2, 36] (Figure 2).

From a further series, the correspondence between results of embryo typing and of typing carried out in foetuses and young calves was 100% (13 couples embryo/calf or foetus with the same typing results). Those first results obtained with biopsies derived from *in vivo* or* in vitro* embryos produced at the research station and biopsies performed in a central laboratory were completed by a set of results where biopsies were made on farm following the collection of *in vivo* produced embryos [34]. Typing was made by using the usual set of 45 micro satellites markers which was completed by the analysis of a complementary set of 13 Single Nucleotide Polymorphism (SNP) markers. From 57 biopsied embryos, the total detection rate was higher for SNPs than for micro satellites (70.2% versus 31.6%; *P* < .01). The detection rates of the markers were not significantly affected by embryo stage, biopsy size, or sex of the embryo. However, from those series of biopsies made under farm conditions by different embryo transfer teams, the proportions of markers detected were much lower than when the biopsies were prepared in the laboratory and immediately followed by whole genome amplification. In addition, despite the amount of preamplified DNA was found sufficient in all samples, the percentage of markers detected varied considerably between teams suggesting that the conditions of preparation and/or transportation may affect the quality of the DNA to be preamplified and consequently the efficiency of the system. To avoid this, conventional freezing of the biopsy cells should be recommended.

Additional experiments were carried out to evaluate and compare the developmental ability of biopsied embryos after *in vitro* culture and the pregnancy rates following transfer of *in vivo* produced embryos previously biopsied and frozen. Embryo survival following the biopsy of *in vitro* produced embryos was not different from the rate observed for nonbiopsied embryos from the same series of production that were used as controls (58/64 versus 18/20; 90%). From subsequent series, the embryonic development *in vitro* following biopsy of *in vivo* and * in vitro *produced embryos was not different (62/70; 89% versus 41/44; 93.2%). These results indicate that the effects of the biopsy by itself on subsequent embryonic development are very limited irrespective of the system used to produce the embryos. 

Pregnancy rates following the transfer on farm of fresh biopsied *in vivo *produced grade 1 and 2 (IETS classification) embryos were over 60% [38, 39] (Table 1). Ponsart et al. [39] reported pregnancy rates of 50% or more following transfer of frozen biopsied embryos on farm and this percentage was close to 60 when transfers were made under the more controlled conditions of a research station (54/90; frozen). In addition, from more recent series in station, it has been shown that grade 3 embryos may be used as well as pregnancy rates following transfer of those were not different when compared to results obtained with grades 1 and 2 [40]. 

When considering these results, the typing from biopsied *in vivo *produced embryos looks realistic as the development rates and pregnancy rates following transfer of biopsied and frozen embryos do not seem to be much affected by the biopsy procedure itself. In addition, those results show that most embryos, even grade 3, could probably be kept in the process and this would allow most of them to be genotyped. Despite the fact that good pregnancy rates have been reported with frozen *in vitro *produced embryos in many countries [1], improvements are probably still necessary for those, because of the selection usually applied before and after freezing by most teams and lack of data on pregnancy rates following direct transfer of large numbers of biopsied and frozen *in vitro *produced embryos.

Calculations have been performed to estimate the genetic and economic advantages of using embryo typing in association with MOET when compared to the use of conventional embryo transfer alone. In a first study, simulations based on the use of the first generation of MAS markers were made from real series of observations obtained from females included as donors in genetic schemes and performances of their sons evaluated at various ages [41]. Those simulations have shown that the use of embryo typing is associated with very significant advantages at the time of early evaluations (up to 1 year of age) that disappears at the time of final evaluation. This indicates that when using this first generation of typing method (limited number of micro satellites), the embryo typing scenario suffered both from the lack of precision of the genetic information from young donor females and from the lack of precision of the genotyping evaluation which was used to select those embryos early in life. These defaults are much less important now and almost disappear today (i) due to the better knowledge associated with young parents facilitated by the accumulation of genetic information through generations and (ii) it will become completely negligible/nonexistent with the gain of precision obtained from the use of the 54 k SNP chip or from future Whole Genome Evaluation. Other types of simulations based on the costs induced by different scenarios to produce the same number of bulls of the same genetic value revealed that substantial gains can be achieved with the help of embryo typing if the whole set of reproductive techniques is well controlled.

Additional economic and genetic simulations should be performed in this new context of using high density markers chips to precisely evaluate the costs and advantages for the genetic schemes of such procedures based on embryo typing. Limitations may be encountered in relation to the technical feasibility of using amplified DNA together with the latest generation of high density marker chips. However, preliminary studies from limited numbers of biopsies and typing have shown that the use of preamplified DNA is compatible with the typing from those chips (Lebourhis et al. 2010, unpublished). This must be verified with the next generation of chips that will include 600 000 markers. Cost efficiency of the whole system must be verified also by simulations made from different scenarios. If needed, alternatives may be found by using other types of chips fully compatible with the analysis of preamplified DNA allowing a prescreening of the embryos at a very low cost before performing full genotyping in calves.

### 3.4. Other Reproductive Techniques

To a certain extent, sperm sexing can help to limit the number of embryos to be produced for this purpose and may be used in combination with in vitro fertilisation and in vitro production (IVF-IVP) procedures. Use of semen sexing in association with IVF-IVP may also avoid some of the present limitations of the use of semen sexing in selection schemes in relation to the high number of sperm that must be discarded and the large individual variation associated with the sexing process by flow cytometry [42]. 

Finally, considering the need to maximize genetic variability and due to strong limitations in reproductive efficiency, cloning is unlikely, at least at present, to represent a useful tool in the framework of selection schemes. However, besides selection schemes driven by breeding associations/companies, individual farmers, that may get access to genomic selection, may be interested in the duplication of their best animals with the help of cloning for commercial purposes in countries allowing the use of this process. Some applications may result from the use of transgenesis associated with cloning; however, such technical options especially in the EU context will face strong limitations in relation to ethics, public concern, and political attitude that will probably limit their use to types of production different from agronomics.

## 4. Impact of Genomic Selection on the Use of Reproductive Techniques and More Specifically ART

### 4.1. Genetic Schemes

Artificial Insemination (AI), Multiple Ovulation and Embryo Transfer (MOET) and/or, depending on legislation in individual EU countries, Ovum Pick Up associated with *in vitro* Embryo Production (OPU-IVP) have already been used in the past to generate the future sires to be widely used following selection through highly effective but very costly progeny testing programmes. The changes in breeding strategies and use of reproductive techniques associated with the needs of genomic selection are on the way. They result from the organisation of selection schemes which are already today completely different. As shown before, efficiency of embryo transfer, OPU and IVF, will be critical and these techniques will be probably more used than in the past to increase the number of candidates. There is a large phenotypic variability between individual females for in vivo and in vitro productions [43, 44]. Taking into account the genetic index of donor females in OPU-IVP production may also be used to optimize the results [44]. On top of this, the potential value of the genotyped animals will probably lead breeding associations/companies to adopt strategies allowing them to control the production of genome-selected animals. This will lead them to reinforce the use of embryo-based reproductive techniques MOET and IVP in nucleus herds to give birth to previously (pre) selected animals within a given structure/company and not on farm. In this context, the success of embryo typing before transfer may be more and more critical for the breeding organisations and some of the companies involved in Eurogenomics have already started to include embryo typing in their selection process.

### 4.2. Commercial Activity

As soon as genotyping will be extended, farmers will have access to the corresponding information in females. This will probably induce a strong rise in the demand for ET and even OPU and IVF from farmers wishing to optimize the value of their best females within their herds and/or for commercial purposes.

## 5. Conclusion

In the new context of genomic selection, there is still a lot of work for the reproductive physiologist to study gene expression and identify markers and networks of genes associated with fertility. As far as selection for fertility is concerned, more precise phenotyping is needed for particular reproductive events and more especially for precocity of reproductive traits that has not been well characterized so far. More generally, for all production traits and functional traits, in the present context showing very impressive improvements induced by the intensive use of MAS, it is likely that the use of a set of intensive reproductive techniques together with embryo typing will bring very significant advantages to breeding organisations capable of monitoring all those techniques with efficiency. However, strategies must be developed to use all these techniques in such a way that they contribute to maintain genetic variability. It is clear that the emergence of the new methods for genomic selection makes all improvements related to embryo production *in vivo *or *in vitro *and associated techniques very attractive for breeding organisations and companies willing to exploit as much as possible the advantages of genomic selection. There will probably also be some changes in relation to commercial activity due to valuable genomic information becoming available in females that may lead individual farmers/companies to make a larger use of semen sexing and embryo related technologies.

## Figures and Tables

**Table 1:** Pregnancy rates following transfer of biopsied embryos on farms in different French programs since 2005.

Author	Year of transfer	Type of embryos	*N*	Pregnancy rate
Ponsart et al. 2008 [39]	2002–2007	Fresh	1333	63.3%
		Frozen	669	52.0%
Lacaze et al. 2008 [38]	2005–2008 (Aubrac)	Frozen	132	55.3%
